# Physiologic Tailoring of Treatment in Resistant Hypertension

**DOI:** 10.2174/157340310791162695

**Published:** 2010-05

**Authors:** J. David Spence

**Affiliations:** Stroke Prevention & Atherosclerosis Research Centre, 1400 Western Road, London, Ontario, Canada N6G 2V2

**Keywords:** Resistant hypertension, renin, amiloride, primary hyperaldosteronism, renal sodium channel, African-American, Stroke belt.

## Abstract

Resistant hypertension is a major opportunity for prevention of cardiovascular disease. Despite widespread dissemination of consensus guidelines, most patients are uncontrolled with approaches that assume that all patients are the same.

Causes of resistant hypertension include 1) non-compliance 2) consumption of substances that aggravate hypertension (such as salt, alcohol, nonsteroidal anti-inflammatory drugs, licorice, decongestants) and 3) secondary hypertension.

Selecting the appropriate therapy for a patient depends on finding the cause of the hypertension. Once rare causes have been eliminated (such as pheochromocytoma, licorice, adult coarctation of the aorta), the cause will usually be found by intelligent interpretation (in the light of medications then being taken) of plasma renin and aldosterone.

If stimulated renin is low and the aldosterone is high, the problem is primary aldosteronism, and the best treatment is usually aldosterone antagonists (spironolactone or eplerenone; high-dose amiloride for men where eplerenone is not available). If the renin is high, with secondary hyperaldosteronism, the best treatment is angiotensin receptor blockers or aliskiren. If the renin and aldosterone are both low the problem is over-activity of renal sodium channels and the treatment is amiloride. This approach is particularly important in patients of African origin, who are more likely to have low-renin hypertension.

Uncontrolled hypertension represents a failure of the usual approach of evidence-based guidelines. Despite regular updating and wide dissemination, even the best consensus guidelines achieve abysmal results. In Canada, where the guidelines are admirably administered and updated annually, the proportion of hypertensive patients controlled to <140/90 has failed to increase from the 17% controlled in 1997 [1] to the 15.8% controlled in 2007 [2]. (This may have changed recently.) Control rates have been somewhat better (29%) in the United States, and even worse (< 10%) in Europe [3]. In my view, a major reason for the failure of such guidelines is that they tend to make the fallacious assumption that all patients should be treated the same way, with insufficient attention to tailoring the therapy to the cause of hypertension in patients with resistant hypertension.

The consequences of this failure are enormous, and represent a major opportunity for prevention of the consequences of hypertension, particularly stroke. A particular tragedy is the failure of physicians to recognize that patients of African origin are more likely to have specific causes of low-renin hypertension that require specific therapies [4, 5]. This probably accounts for the observation by Howard *et al*. [6] that black patients in the stroke belt are more likely to be aware of and more likely to be treated for hypertension, more likely to be treated more intensively, but less likely to be controlled. The result of this is that African-Americans have twice the risk of stroke[7]; the tragedy is that this is preventable. 

## CAUSES OF RESISTANT HYPERTENSION

The causes of resistant hypertension are summarized in Table **1**. Non-compliance with medication is common, and approximately half of patients will admit to non-compliance [8]. Compliance is better with medications that have less adverse effects [9, 10]. Followup with pharmacists may be needed to determine if prescriptions have been filled. If the patient is taking the prescribed medication, a careful history is required to determine if the blood pressure is being aggravated by consumption of substances such as excess salt, licorice, alcohol, non-steroidal anti-inflammatory drugs, decongestants or oral contraceptives. (Sulindac, which spares renomedullary prostaglandins, is the only NSAID that does not aggravate hypertension [11]. Naproxcinod shows promise for the future [12]. ) If none of these causes is apparent, the patient requires investigation for secondary hypertension.

Some 90% of strokes among hypertensive patients are attributable to uncontrolled hypertension [13]. In London, Ontario, a concerted effort to increase detection and treatment of hypertension reduced stroke by half, between 1978 and 1983 [14]. This was accomplished by a large effort on the part of the Department of Family Medicine [15, 16], in concert with a Hypertension Clinic using stimulated plasma renin levels [17] to detect causes of secondary hypertension. In 20 years [18] I found only 52 patients with pheochromocytoma, 9 from licorice, 9 from hypernephroma, and 3 adult aortic coarctations. Since then we have seen one case of atherosclerotic occlusion above and below the renal arteries, mimicking bilateral renal artery stenosis [19]. 

**Fig. (1). Central role of the renin-angiotensin-aldosterone axis in resistant hypertension:**
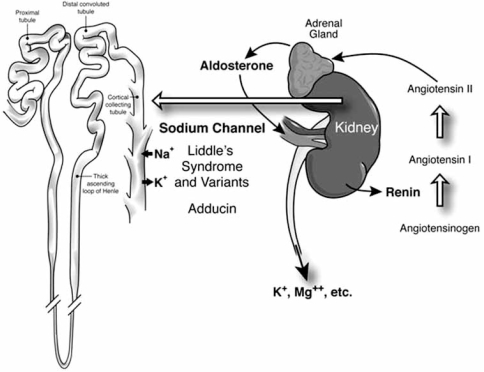
In normal homeostasis, renin is released under conditions of low blood pressure or dehydration; that activates aldosterone release, which causes salt and water retention, and excretion of potassium, magnesium, zinc and other ions. Disorders of this physiology can cause hypertension with three patterns of renin and aldosterone levels: Primary hyperaldosteronism causes salt and water retention, feeding back to suppress renin. Renal or renovascular causes of hypertension cause elevated renin with secondary hyperaldosteronism. Abnormalities of the renal tubular epithelial sodium channel (Liddle’s syndrome and other polymorphisms of the renal sodium channel, or adducin), cause salt and water retention and suppress both renin and aldosterone

Once those rare causes of hypertension are eliminated, the remainder are to be found in the renin-angiotensin-aldosterone axis, or in abnormalities of the renal tubular sodium channel (Fig. **1**). Patients with high levels of renin and secondary hyperaldosteronism require investigation for renal or renovascular causes, and may need renal decompression (for example, relief of a ureteric stricture) or revascularization [20]. Their medical therapy is primarily with angiotensin receptor antagonists (ARBs) or aliskiren. (Inhibitors of angiotensin converting enzyme are not as effective because of angiotensin II escape pathways such as chymase and cathepsin; they may act mainly by increasing nitric oxide release *via* blockade of bradykinin inactivation, and can be thought of as similar to long-acting nitrates.) Patients with low renin and high levels of aldosterone have primary aldosteronism, which accounts for 20% of resistant hypertension [21]. It is crucial to recognize that the imperative to find the cause of resistant hypertension is not mainly about finding cases for surgeons, it is about choosing appropriate medical therapy [22]; this is particularly important in patients of African origin.

## PATIENTS OF AFRICAN ORIGIN

Although most US physicians are aware that African-American patients have lower levels of plasma renin on average, seldom are the reasons given sufficient consideration. There are important lessons to be learned from Sub-Saharan Africa [5]. Rayner and colleagues have found in the Khoi San people (indigenous people of Southern Africa, thought to be candidates for the original homo sapiens) that 20% have a polymorphism of the renal tubular epithelial sodium channel (a variant of Liddle’s syndrome) that causes salt and water retention and hypertension (personal communication, 2009). Though this ability to retain salt and water may have been adaptive to survival in the Kalahari Desert, it causes severe hypertension in conditions of abundance of salt and water. This polymorphism accounts for 6% of hypertension in South African blacks [23], and a related polymorphism was found by the group of McGregor in London, UK, in 6% of black patients (mostly from the Caribbean) [24, 25]. These conditions are specifically treated with amiloride [24]. (Although thiazide and other diuretics may lower blood pressure in these conditions, they would be more likely to aggravate potassium depletion.)

In addition to the selective advantage of salt and water retention for survival in Sub-Saharan Africa, there may have been additional selective pressure favouring salt and water retention (and therefore low-renin hypertension) in the Atlantic crossing on slave ships, and then for survival working in the cotton fields [26].

Besides variants of the renal sodium channel, patients of African origin are more likely to have primary aldosteronism due to bilateral hyperplasia of the adrenal cortex [27]. It is increasingly recognized that solitary adrenal nodules that are surgically curable represent a shrinking proportion of primary aldosteronism, and that therapy is medical in most cases. Biglieri *et al.* first described four cases of primary adrenocortical hyperplasia in 1984 [28]. At that time we had already identified approximately 70 cases, of which 10 required adrenalectomy because they could not be controlled medically. Of those 10 cases 4 were black (whereas less than 1% of our patients were black), suggesting a major disproportion of this condition among black patients. One of these was from Swaziland; the other 3 came from North Buxton, a black community in Southwestern Ontario, descended from escaped slaves who came to Canada *via* the Underground Railroad.

## ADRENOCORTICAL HYPERPLASIA IS USUALLY BILATERAL

Between 1980 and 1983, with Dr. Albert Driedger, we studied 100 patients with stimulated plasma renin levels <1ng/ml/hr. with iodocholesterol scanning before and after dexamethasone suppression. We were looking for patients with unilateral adenomas, who might benefit from adrenalectomy. To our astonishment, not a single case had a unilateral hot adrenal gland. Approximately 70% had bilateral hot adrenals, and 30% were normal. I suspect that some or many of the normal cases were missed cases of Liddle’s syndrome and variants, as in those days we did not have access to measurement of plasma aldosterone. In our experience, therefore, most primary aldosteronism is due to bilateral hyperplasia.

Two forms of familial primary aldosteronism have so far been reported; in both these conditions nodular hyperplasia, with nodules large enough to resemble (and be confused with) adenomas, is common [29]. The first was dexamethasone-suppressible hypertension, first described by Laidlaw’s group [30], and then elegantly elucidated by Lifton *et al.* to be due to a chimeric gene; a fusion of the 5’ regulatory region of 11-B hydroxylase to aldosterone synthase, with the result that aldosterone is driven by ACTH [31]. This condition is treated with low-dose dexamethasone. The second is not dexamethasone-suppressible, and is linked to 7p22 [29, 32]. 

Table **2** lists some of the causes of low-renin hypertension.

Table **3** shows our algorithm for sorting out the cause and the primary treatment for patients with resistant hypertension, once rare causes such as pheochromocytoma, licorice and aortic coarctation are excluded. Plasma renin and aldosterone are best measured in a stimulated condition, as described by Dawson’s group [41]. If the patient is already taking medication that stimulates renin, such as diuretics, ACE inhibitors or ARB’s, then further stimulation is not needed. However, the results must be interpreted in the light of the medication being taken. ARB’s (and probably aliskiren) are particularly effective at suppressing aldosterone production, so a patient with a low or low-normal renin level and a high or high-normal aldosterone level while taking an ARB probably has primary aldosteronism, for the purposes of adjusting medical therapy. Further investigation would be needed to justify adrenalectomy.

## SOME PATIENTS HAVE MORE THAN ONE FORM OF SECONDARY HYPERTENSION

Not included in the algorithm are the very rare cases with more than one cause of hypertension. In 20 years as the physician of last resort for resistant hypertension for a catchment area with a population of approximately 2 million (and with some referrals from far beyond), I saw about 15 patients with primary aldosteronism who had gone on to develop renal artery stenosis. These patients thus had both primary and secondary hyperaldosteronism, and required both aldosterone antagonists and ARBs (a combination that would usually be avoided because of the risk of hyperkalemia). One patient of mine with both primary and secondary hyperaldosteronism has primary aldosteronism and polycystic kidneys, with plasma aldosterone levels that are 50 times the upper limit of normal. This is a rare but recognized syndrome; there is some evidence that potassium depletion from primary aldosteronism exacerbates renal cysts [42, 43]. It seems likely that some patients with Liddle’s variants might also have other causes of secondary hypertension. Such cases require thoughtful interpretation of all the clinical data including the levels of renin and aldosterone, and the medications being taken at the time of renin and aldosterone sampling.

Egan *et al.* [44] recently published a randomized trial showing that measurement of plasma renin improved therapy of resistant hypertension. In my view it is better to measure both renin and aldosterone so that renal tubular sodium channel variants and other factors affecting the function of the renal sodium channel (adducin [38], endogenous ouabain [39]) can be differentiated from primary aldosteronism. The reason this is important is that medical therapy for primary aldosteronism is best with aldosterone antagonists (spironolactone, or for men, eplerenone), because they counteract the other adverse effects of spironolactone on the vasculature beyond elevation of blood pressure: inflammation, remodelling and fibrosis [32, 34, 45], On the other hand, salt and water retention based on enhanced function of the sodium channel, with low levels of renin and aldosterone, are best treated with amiloride (or possibly rostafuroxin [38]). The appropriate selection of amiloride or aldosterone antagonists leads not only to more physiological therapy, but may also improve adherence, by preventing the adverse effects of kaliuretic diuretics.

## SUMMARY AND CONCLUSIONS

Uncontrolled hypertension is a major preventable cause of stroke, renal failure, heart failure and dementia. To reduce the burden of this problem it is important to identify the underlying cause of resistant hypertension so that the appropriate therapy can be selected. Once rare causes of hypertension have been excluded, measuring the levels of plasma renin and aldosterone will identify the cause in most cases, for purposes of medical therapy. The most neglected causes of resistant hypertension, which particularly afflict patients of African origin, are primary aldosteronism (usually due to bilateral hyperplasia), best treated with aldosterone antagonists, and variants of the renal tubular sodium channel, which are specifically treated with amiloride.

## Figures and Tables

**Table 1:** Causes of Resistant Hypertension

Non-compliance Half of patients will admit it [8] Better with drugs that have less adverse effects [9, 10] Consumption of substances that aggravate hypertension Salt, licorice, NSAID’s, oral contraceptives, decongestants Sulindac is the only NSAID that doesn’t raise blood pressure [11] Secondary hypertension Most neglected causes are primary hyperaldosteronism due to adrenocortical hyperplasia, and variants of the renal tubular sodium channel

**Table 2:** Causes of Low-Renin Hypertension

Conn’s syndrome (unilateral surgically curable adenoma – if it exists)
Primary adrenocortical hyperplasia [5, 27, 28]
Familial hyperaldosteronism
Type I Dexamethasone-suppressible hypertension [30, 33]; chimeric gene causing ACTH-dependent aldosterone production
Treat with low-dose dexamethasone
Type II Linked to chromosome 7p22 [29]
Treat with aldosterone antagonists [34]; rarely surgical
Gordon’s syndrome [35]
treat with salt restriction
Renal tubular sodium channel mutations or alteration
Liddle’s syndrome [36] and variants [24, 25, 37]
5-6% of HT in blacks low aldo and renin; treat with amiloride
Adducin polymorphisms [38]
Endogenous ouabain [39]
low aldo and renin; treat with amiloride (possibly rostafuroxin)
GIP dependent cortisol excess with nodular hyperplasia [40]

**Table 3:** Physiologic Tailoring of Medical Therapy Based on Plasma Renin and Aldosterone

	Primary Aldosteronism	Liddle’s Variants, Adducin Polymorphisms	Renal or Renovascular

Renin	Low	Low	High

Aldosterone	High	Low	High

Primary treatment	Aldosterone antagonists	Amiloride	Angiotensin receptor blockers
	Spironolactone		Aliskiren
	Eplerenone		
	(Amiloride for men where eplerenone is not available)		
	(Rarely surgical)	(possibly rostafuroxin)	(Revascularization or decompression may be necessary)

## References

[1] Joffres MR, Ghadirian P, Fodor JG, Petrasovits A, Chockalingam A, Hamet P (1997). Awareness, treatment and control of hypertension in Canada. Am J Hypertens.

[2] Petrella RJ, Merikle EP, Jones J (2007). Prevalence, treatment, and control of hypertension in primary care: gaps, trends, and opportunities. J Clin Hypertens (Greenwich ).

[3] Wolf-Maier K, Cooper RS, Kramer H (2004). Hypertension treatment and control in five European countries, Canada, and the United States. Hypertension.

[4] Lindhorst J, Alexander N, Blignaut J, Rayner B (2007). Differences in hypertension between blacks and whites: an overview. Cardiovasc J Afr.

[5] Opie LH, Seedat YK (2005). Hypertension in sub-Saharan African populations. Circulation.

[6] Howard G, Prineas R, Moy C (2006). Racial and geographic differences in awareness, treatment, and control of hypertension: the Reasons for Geographic And Racial Differences in Stroke study. Stroke.

[7] Lloyd-Jones D, Adams R, Carnethon M (2009). Heart Disease and Stroke Statistics-2009 Update. A Report From the American Heart Association Statistics Committee and Stroke Statistics Subcommittee. Circulation.

[8] Haynes RB, Taylor DW, Sackett DL, Gibson ES, Bernholz CD, Mukherjee J (1980). Can simple clinical measurements detect patient noncompliance?. Hypertension.

[9] Bloom BS (1998). Continuation of initial antihypertensive medication after 1 year of therapy. Clin Ther.

[10] Marentette MA, Gerth WC, Billings DK, Zarnke KB (2002). Antihypertensive persistence and drug class. Can J Cardiol.

[11] Wong DG, Spence JD, Lamki L, McDonald JWD (1986). Effect of non-steroidal anti-inflammatory drugs on control of hypertension by beta-blockers and diuretics. Lancet.

[12] White WB, Schnitzer TJ, Fleming R, Duquesroix B, Beekman M (2009). Effects of the cyclooxygenase inhibiting nitric oxide donator naproxcinod versus naproxen on systemic blood pressure in patients with osteoarthritis. Am J Cardiol.

[13] Li C, Engström G, Hedblad B, Berglund G, Janzon L (2005). Blood pressure control and risk of stroke: a population-based prospective cohort study. Stroke.

[14] Spence JD (1986). Antihypertensive drugs and prevention of atherosclerotic stroke. Stroke.

[15] Bass MJ, McWhinney IR, Donner A (1986). Do family physicians need medical assistants to detect and manage hypertension?. Can Med Assoc J.

[16] Birkett NJ, Donner AP, Maynard M (1985). Prevalence and control of hypertension in an Ontario county. Can Med Assoc J.

[17] Wallach L, Nyarai I, Dawson KG (1975). Stimulated renin: a screening test for hypertension. Ann Int Med.

[18] Spence JD (1999). Physiologic tailoring of therapy for resistant hypertension:20 year' experience with stimulated renin profiling. Am J Hypertens.

[19] Hackam DG, Thain LMF, Abassakoor A, McKenzie FN, Spence JD (2001). Trapped renal arteries: Functional renal artery stenosis due to occlusion of the aorta in the arch and below the kidneys. Can J Cardiol.

[20] Spence JD (2002). Treatment options for renovascular hypertension. Expert Opin Pharmacother.

[21] Calhoun DA, Jones D, Textor S (2008). Resistant hypertension: diagnosis, evaluation, and treatment: a scientific statement from the American Heart Association Professional Education Committee of the Council for High Blood Pressure Research. Circulation.

[22] Spence JD (2009). Diagnosis of primary aldosteronism: for medical management, not just surgery. J Hypertens.

[23] Rayner BL, Owen EP, King JA (2003). A new mutation, R563Q, of the beta subunit of the epithelial sodium channel associated with low-renin, low-aldosterone hypertension. J Hypertens.

[24] Baker EH, Duggal A, Dong Y (2002). Amiloride, a specific drug for hypertension in black people with T594M variant?. Hypertension.

[25] Swift PA, MacGregor GA (2004). Genetic variation in the epithelial sodium channel: a risk factor for hypertension in people of African origin. Adv Ren Replace Ther.

[26] Grim CE, Robinson M (2003). Salt, slavery and survival- hypertension in the African diaspora. Epidemiology.

[27] Russell RP, Masi AT (1970). The prevalence of adrenal cortical hyperplasia at autopsy and its association with hypertension. Ann Intern Med.

[28] Biglieri EG, Kater CE, Arteaga EE (1984). Primary aldosteronism is composed of primary adrenal hyperplasia and adenoma. J Hypertens.

[29] Sukor N, Mulatero P, Gordon RD (2008). Further evidence for linkage of familial hyperaldosteronism type II at chromosome 7p22 in Italian as well as Australian and South American families. J Hypertens.

[30] Sutherland DJ, Ruse JL, Laidlaw JC (1966). Hypertension, increased aldosterone secretion and low plasma renin activity relieved by dexamethasone. Can Med Assoc J.

[31] Lifton RP, Dluhy RG, Powers M (1992). A chimaeric 11 beta-hydroxylase/aldosterone synthase gene causes glucocorticoid-remediable aldosteronism and human hypertension. Nature.

[32] Stowasser M (2009). Update in primary aldosteronism. J Clin Endocrinol Metab.

[33] Lifton RP, Dluhy RG, Powers M (1992). Hereditary hypertension caused by chimaeric gene duplications and ectopic expression of aldosterone synthase. Nat Genet.

[34] Janmohamed S, Bouloux PM (2006). The pharmacological treatment of primary aldosteronism. Expert Opin Pharmacother.

[35] Gordon RD, Geddes RA, Pawsey CG, O'Halloran MW (1970). Hypertension and severe hyperkalaemia associated with suppression of renin and aldosterone and completely reversed by dietary sodium restriction. Australas Ann Med.

[36] Liddle GW, Bledsoe T, Coppage WS (1963). A familial renal disorder simulating primary aldosteronism but with negligable aldosterone secretion. Trans Assoc Am Physicians.

[37] Warnock DG (2001). Liddle syndrome: genetics and mechanisms of Na+ channel defects. Am J Med Sci.

[38] Manunta P, Bianchi G (2006). Pharmacogenomics and pharmacogenetics of hypertension: update and perspectives--the adducin paradigm. J Am Soc Nephrol.

[39] Ferrandi M, Molinari I, Bianchi G, Ferrari P (2006). Ouabain-dependent signaling in caveolae as a novel therapeutic target for hypertension. Cell Mol Biol (Noisy -le-grand).

[40] Lacroix A, Bolte E, Tremblay J (1992). Gastric inhibitory polypeptide-dependent cortisol hypersecretion-a new cause of Cushing's syndrome. N Engl J Med.

[41] Wallach L, Nyarai I, Dawson KG (1975). Stimulated renin: a screening test for hypertension. Ann Intern Med.

[42] Torres VE, Young WF Jr, Offord KP, Hattery RR (1990). Association of hypokalemia, aldosteronism, and renal cysts. N Engl J Med.

[43] Novello M, Catena C, Nadalini E (2007). Renal cysts and hypokalemia in primary aldosteronism: results of long-term follow-up after treatment. J Hypertens.

[44] Egan BM, Basile JN, Rehman SU (2009). Plasma Renin test-guided drug treatment algorithm for correcting patients with treated but uncontrolled hypertension: a randomized controlled trial. Am J Hypertens.

[45] Brown NJ (2008). Aldosterone and vascular inflammation. Hypertension.

